# Bromodomain-containing protein 4 silencing by microRNA-765 produces anti-ovarian cancer cell activity

**DOI:** 10.18632/aging.202632

**Published:** 2021-03-03

**Authors:** Yong-Jun Ji, Yang Shao, Jie Zhang, Xu Zhang, Ping Qiang

**Affiliations:** 1 Obstetrics and Gynecology Department, Suzhou Ninth People's Hospital of Soochow University, Suzhou, China; 2 Obstetrics and Gynecology Department, The Affiliated Zhangjiagang Hospital of Soochow University, Zhangjiagang, China

**Keywords:** ovarian cancer, BRD4, miRNA

## Abstract

Bromodomain-containing protein 4 (BRD4) overexpression promotes ovarian cancer progression, and represents an important therapeutic oncotarget. This current study identified microRNA-765 (miR-765) as a novel BRD4-targeting miRNA. We showed that miR-765 directly associated with and silenced BRD4. In primary ovarian cancer cells and established cell lines (SKOV3 and CaOV3), ectopic overexpression of miR-765 inhibited cancer cell proliferation, migration and invasion, and induced apoptosis activation. In contrast, miR-765 inhibition by its anti-sense induced BRD4 upregulation to promote ovarian cancer cell proliferation, migration and invasion. Significantly, miR-765 overexpression-induced anti-ovarian cancer cell activity was largely attenuated by restoring BRD4 expression through an UTR-null BRD4 construct. Moreover, CRISPR/Cas9-induced BRD4 knockout (KO)inhibited proliferation and activated apoptosis in ovarian cancer cells. BRD4 KO in ovarian cancer cells abolished the functional impact of miR-765. miR-765 expression levels were downregulated in human ovarian cancer tissues and cells, correlating with the upregulation of *BRD4* mRNA. Collectively, BRD4 silencing by miR-765produces significant anti-ovarian cancer cell activity. miR-765 could be further tested for its anti-ovarian cancer potential.

## INTRODUCTION

Ovarian cancer is the seventh most common cancer and the eighth most common cause of cancer-related mortalities among women [1, 2]. It is estimated that each year there are over 1.2 million new cases of ovarian cancers and 160,000 deaths around the world [1, 2]. The majority of ovarian cancer patients are diagnosed at the late stages, possibly due to a lack of typical clinical symptoms or early screening methods [3–5]. The current therapies for this devastating disease are surgery and/or platinum-based chemotherapy [3–5]. As a result of profound resistance to current available therapies, studies testing agents targeting novel cell signaling pathways and oncogenic genes required for ovarian cancer tumorigenesis and progression are necessary [3–5].

The bromodomain and extraterminal (BET) family chromatin reader proteins have five primary members: bromodomain-containing protein 1 (BRD1), BRD2, BRD3, BRD4 and BRDT [6, 7]. BET proteins share two conserved N-terminal bromodomains that can bind to histones’ N-acetyl lysine residues [6, 7]. Among them, BRD4directly binds to the mediator complex and positive transcription elongation factor (pTEFb) to promote RNA-pol II-mediated elongation and transcription [8, 9]. Moreover, BRD4 interacts with the acetylated transcription factors (RelA, ERα, TWIST and many others) to promote expression of multiple key oncogenic genes [10, 11] including c-Myc, Bcl-2, FoxM1(Forkhead box protein M1) and cyclin D1 [6, 7, 11, 12].

A shRNA screening study revealed that BRD4 is a promising therapeutic target of ovarian cancer [13]. BRD4 is overexpressed in ovarian cancer, required for proliferation and survival of established ovarian cancer cell lines and primary cancer cells [13]. BRD4 inhibition using small-molecule BET inhibitors (JQ1 and I-BET151) induced robust and broad anti-ovarian cancer cell activity [14]. Therefore, targeting BRD4 could be an important anti-ovarian cancer therapy.

MicroRNAs (miRNAs, 21–25 nucleotide long) and non-coding RNAs (ncRNAs) are able to effectively alter gene expression at translational and/or post-transcriptional levels [15–18]. By binding to the 3’ untranslated region (3’-UTR) of the complementary mRNAs, miRNAs would cause translation inhibition and/or mRNA degradation of targeted genes [15–18]. Dysregulation of miRNA, which is linked to tumorigenesis and cancer progression, has emerged as a novel characteristic marker of ovarian cancer [19–21]. The present study identified microRNA-765 (miR-765) as a novel BRD4-targeting miRNA. miR-765-induced BRD4 silencing is able to produce robust anti-ovarian cancer cell activity.

## RESULTS

### miR-765 associates with and silences BRD4 in ovarian cancer cells

**Figure 1:**
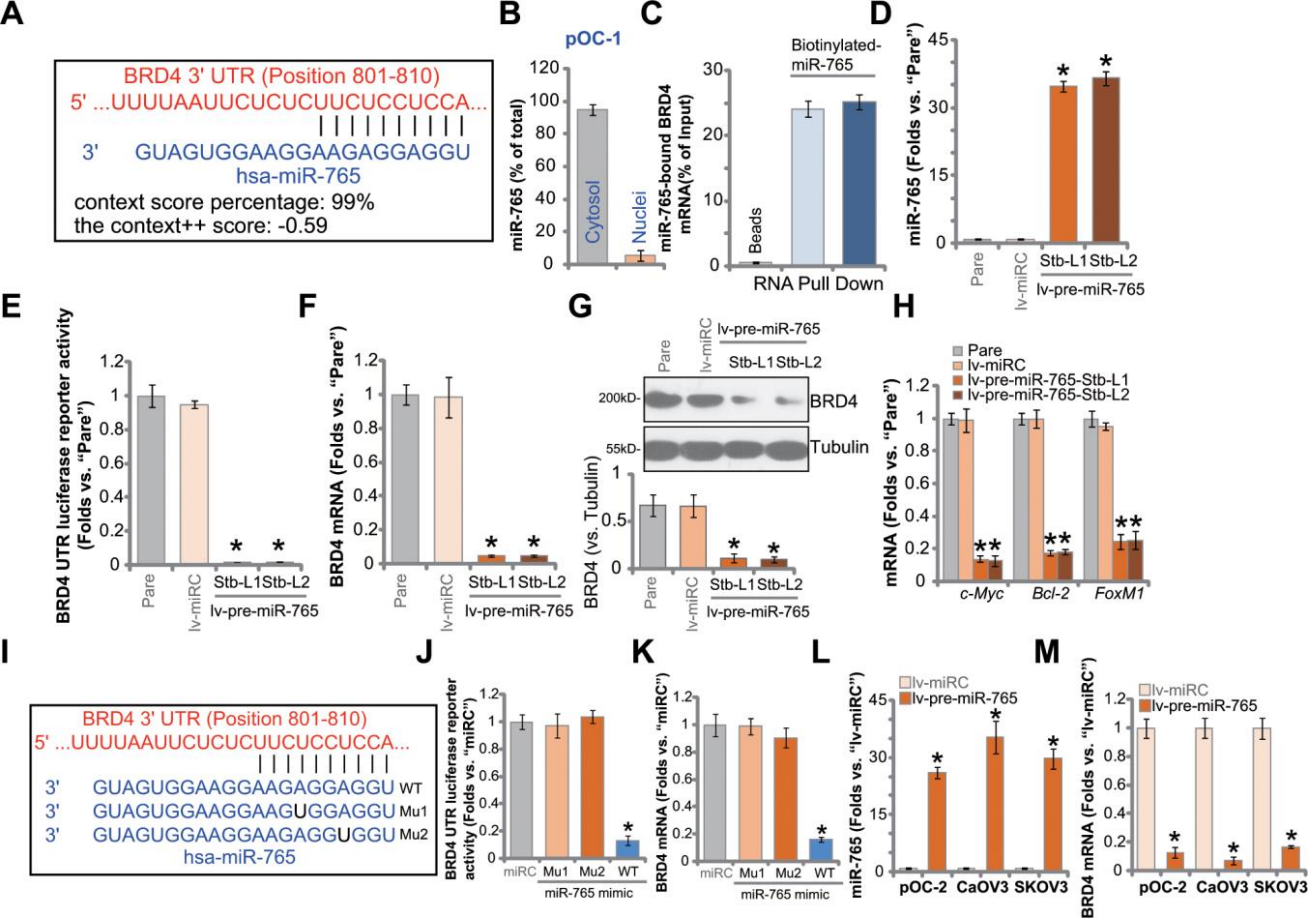
**miR-765 associates with and silences BRD4 in ovarian cancer cells.** MicroRNA-765 (miR-765) putatively binds to *BRD4* 3'-UTR (untranslated region) at position 801-810. (**A**) Subcellular expression (in cytosol and nuclear fractions) of endogenous miR-765 in pOC-1 primary ovarian cancer cells. (**B**) RNA pull-down assay results suggested directing binding between biotinylated-miR-765 and *BRD4* mRNA in pOC-1 cells (**C**); the primary ovarian cancer cells (pOC-1 and pOC-2) (**D**–**H**) or the established cell lines (CaOV3 and SKOV3) (**L**, **M**) were transduced with the lentiviral construct encoding pre-miR-765 sequence (lv-pre-miR-765) or scramble non-sense miRNA (lv-miRC). After selection by puromycin stable cells were established, expression of miR-765 and listed genes was tested by qPCR and Western blotting assays (**D**, **F**–**H**, **L**, **M**), and results quantified; the *BRD4* 3’-UTR luciferase reporter activity was tested as well (**E**). The pOC-1 cells were transfected with the wild-type (WT) or the mutant miR-765 mimic (sequences listed in **I**), at 500 nM each for 36h, the relative BRD4 UTR luciferase reporter activity (**J**) and *BRD4* mRNA expression (**K**) were shown. “Pare” stands for the parental control cells. “miRC” stands for microRNA control mimic. For each assay, n=5 (five replicate well/dishes). Data were presented as mean ± standard deviation (SD). * *p* < 0.05 vs. “lv-miRC”/“miRC” cells. Experiments in this figure were repeated five times with similar results obtained.

The miRNA database TargetScan (V7.2) [22] was searched to explore possible miRNAs putatively binding *BRD4* 3’-UTR [22]. Eleven (11) of these miRNAs showed a context^++^ score less than -0.5 and context^++^ score percentile over 99% (generated from TargetScan). It indicated a high percentage of direct binding between these proposed miRNAs and *BRD4* 3’-UTR [22]. Next, each of the 11 miRNA mimics (500 nM for 36h) was transfected to SKOV3 ovarian cancer cells. The preliminary results found one particular miRNA, microRNA-765 (miR-765), with the most dramatic *BRD4* mRNA silencing. As shown, miR-765 putatively binds to *BRD4* 3’-UTR (at position 801-810) (Figure 1A). The context^++^ score is -0.59 and the context^++^ score percentile is 99% (Figure 1A). Subcellular miR-765 distribution assay confirmed that miR-765 mainly located at the cytosol of primary human ovarian cancer cells, pOC-1 (Figure 1B). By employing a RNA pull-down assay, we found that biotinylated-miR-765 directly associated with *BRD4* mRNA in pOC-1 cells (Figure 1C).

To study whether miR-765 could alter *BRD4* expression, a lentiviral construct encoding miR-765 precursor sequence, lv-pre-miR-765, was transduced to pOC-1 cells. Following selection by puromycin, two stable cell lines, lv-pre-miR-765-Stb-L1 and lv-pre-miR-765-Stb-L2, were established. Analyzing mature miR-765 expression via qPCR assays, we found that miR-765 levels increased over 35 folds in lv-pre-miR-765-expressing cells (Figure 1D). Importantly, in pOC-1 cells, lv-pre-miR-765 potently decreased BRD4 3’-UTR luciferase reporter activity (Figure 1E). In addition, *BRD4* mRNA expression reduced over 90% in the miR-765-overexpresed stable pOC-1 cells (Figure 1F). As a result, BRD4 protein was downregulated as well (Figure 1G). mRNA expression of BRD4-dependent genes, including *c-Myc*, *Bcl-2* and *FoxM1*, was reduced in miR-765-overexpressed cells (Figure 1H). Therefore, ectopic overexpression of miR-765 silenced BRD4 in pOC-1 ovarian cancer cells. The lentiviral construct encoding the non-sense miRNA control, or lv-miRC, did not alter miR-765-BRD4 expression in pOC-1 cells (Figure 1D–1H).

To further support a direct binding between miR-765 and *BRD4* mRNA, two mutant miR-765 mimics, namely “Mu1” and “Mu2”, were generated. The two mutants contain mutations at proposed bindings sites to *BRD4* 3’-UTR (Figure 1I). The wild-type (WT) or the mutant mimics were separately transfected to pOC-1 cells. Only WT miR-765 mimic resulted in significant reductions in BRD4 3’-UTR luciferase reporter activity (Figure 1J) as well as *BRD4* mRNA expression (Figure 1K), while the two mutants were completely ineffective (Figure 1J, 1K). In other ovarian cancer cells, including pOC-2 primary cells and established cell lines (SKOV3 and CaOV3), stable transfection of lv-pre-miR-765 similarly resulted in robust miR-765 upregulation (Figure 1L) and *BRD4* mRNA downregulation (Figure 1M). These results together indicated that miR-765 directly associates with and silences BRD4 in ovarian cancer cells.

### Ectopic overexpression of miR-765 induces significant anti-ovarian cancer cell activity

**Figure 2:**
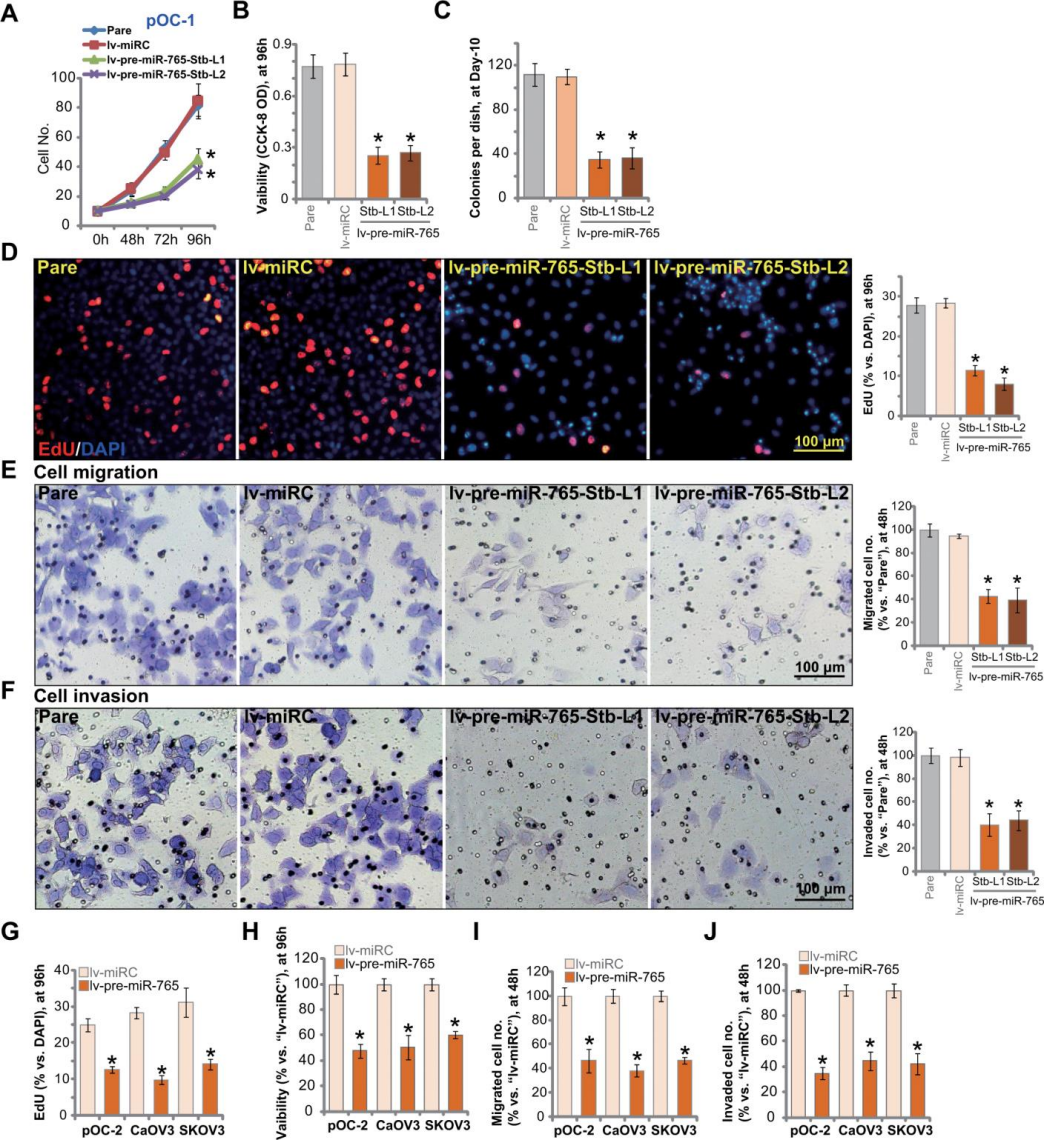
**Ectopic overexpression of miR-765 induces significant anti-ovarian cancer cell activity.** Primary ovarian cancer cells (pOC-1 and pOC-2) (**A**–**J**) or established cell lines (CaOV3 and SKOV3) (**G**–**J**) were transduced with the lentiviral construct encoding pre-miR-765 sequence (lv-pre-miR-765) or scramble non-sense miRNA (lv-miRC). After selection by puromycin stable cells were established. Cells were further cultured for applied time periods, cell growth (**A**), cell viability (CCK-8 OD, **B**, **H**), colony formation (**C**) and proliferation (by counting EdU-positive nuclei ratio, **D**, **G**), as well as cell migration (“Transwell” assays, **E**, **I**) and invasion (“Matrigel Transwell” assays, **F**, **J**) were tested, and results quantified. “Pare” stands for the parental control cells. For each assay, n=5 (five replicate well/dishes). Data were presented as mean ± standard deviation (SD). * *p* < 0.05 vs. “lv-miRC” cells. Experiments in this figure were repeated five times with similar results obtained. Scale bar=100 μm (**D**–**F**).

Results in Figure 1 suggested that miR-765 is a potential BRD4-targeting miRNA. Since BRD4 is an important therapeutic target of ovarian cancer, we next tested whether miR-765 could alter ovarian cancer cell functions. Cell growth curve results in Figure 2A demonstrated that the growth of miR-765-overexpressed pOC-1 cells, lv-pre-miR-765-Stb-L1/L2 (see Figure 1), was significantly slower than the control pOC-1 cells. When testing cell viability using CCK-8 assays, we demonstrated that overexpression of miR-765 inhibited the viability of pOC-1 cells (Figure 2B). Moreover, colony formation assay results in Figure 2C showed that the number of viable pOC-1 cell colonies was decreased in lv-pre-miR-765-expressing pOC-1 cells. These results supported the anti-survival activity by miR-765 overexpression.

EdU incorporation was tested to quantitatively measure cell proliferation. As shown, in pOC-1 cells, the EdU-positive nuclei ratio was largely decreased following miR-765 overexpression (Figure 2D). To test pOC-1 cell migration *in vitro*, “Transwell” assays were performed. Results showed that ectopic miR-765 overexpression inhibited pOC-1 cell migration. As the number of migrated cells was significantly decreased in miR-765-overexpressed pOC-1 cells (Figure 2E). Furthermore, pOC-1 cell invasion, tested by “Matrigel Transwell” assays, was largely inhibited as well (Figure 2F). These results showed that ectopic overexpression of miR-765 inhibited pOC-1 cell growth, proliferation, migration and invasion.

Next we tested the potential effect of miR-765 overexpression on oncogenic behaviors in other ovarian cancer cells, including pOC-2 primary cells and established cell lines (SKOV3 and CaOV3). EdU staining assay results in Figure 2G indicated that miR-765 overexpression by lv-pre-miR-765 (see Figure 1) potently inhibited proliferation of the primary and established ovarian cancer cells. Furthermore, CCK-8 viability was decreased in lv-pre-miR-765-expressing ovarian cancer cells (Figure 2H). In pOC-2 cells and established cell lines, ectopic overexpression of miR-765 largely inhibited cell migration and invasion, tested by “Transwell” (Figure 2I) by “Matrigel Transwell” (Figure 2J) assays, respectively. These results further supported that miR-765 overexpression produced significant anti-ovarian cancer cell activity. As expected, the non-sense miRNA control (lv-miRC) did not alter ovarian cancer cell behaviors (Figure 2A–2J).

### Ectopic overexpression of miR-765 induces apoptosis activation in ovarian cancer cells

**Figure 3:**
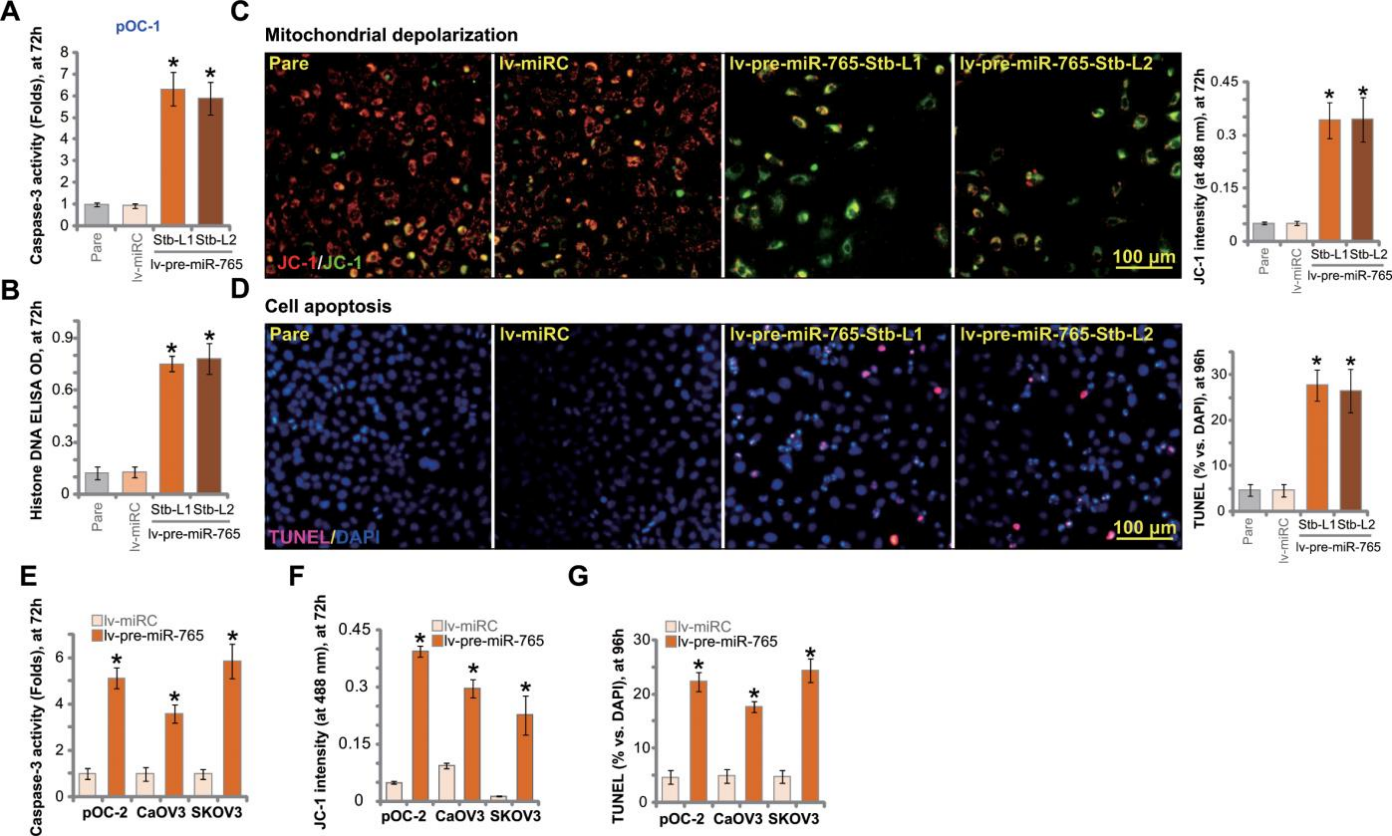
**Ectopic overexpression of miR-765 induces apoptosis activation in ovarian cancer cells.** Primary ovarian cancer cells (pOC-1 and pOC-2) (**A**–**F**) or established cell lines (CaOV3 and SKOV3) (**E**–**G**) were transduced with the lentiviral construct encoding pre-miR-765 sequence (lv-pre-miR-765) or scramble non-sense miRNA (lv-miRC). After selection by puromycin stable cells were established. Cells were further cultured for applied time periods, caspase-3 activation (**A**, **E**). Histone-bound DNA contents (ELISA assays, **B**) and mitochondrial depolarization (by measuring JC-1 green monomer intensity, **C**, **F**) were tested. Cell apoptosis was tested by TUNEL-nuclei staining assay (**D**, **G**). “Pare” stands for the parental control cells. For each assay, n=5 (five replicate well/dishes). Data were presented as mean ± standard deviation (SD). * *p* < 0.05 vs. “lv-miRC” cells. Experiments in this figure were repeated five times with similar results obtained. Scale bar=100 μm (**C**, **D**).

BRD4 inhibition or silencing should be capable of inducing apoptosis in ovarian cancer cells [13, 14]. Results in Figure 2 showed that ectopic overexpression of miR-765 inhibited ovarian cancer cell viability and proliferation. We therefore tested whether miR-765 could induce apoptosis activation. In miR-765-overexpressed pOC-1 cells (lv-pre-miR-765-Stb-L1/L2) the caspase-3 activity was significantly higher than that in control pOC-1 cells (Figure 3A). In addition Histone-bound DNA contents were significantly increased in miR-765-overexpressed pOC-1 cells, indicating accumulation of DNA breaks (Figure 3B).

In apoptotic cells with mitochondrial depolarization, JC-1 fluorescence dye can aggregate to decompose into a monomer form, and the emitted fluorescence shall change from red to green. As demonstrated, there was accumulation of JC-1 green monomers in lv-pre-miR-765-expressing pOC-1 cells, indicating mitochondrial depolarization (Figure 3C). To confirm cell apoptosis activation, we showed that the ratio of TUNEL-positive nuclei was significantly increased in miR-765-overexpressed pOC-1 cells (Figure 3D).

In pOC-2 primary cells and established cell lines (SKOV3 and CaOV3), ectopic overexpression of miR-765 by lv-pre-miR-765 potentiated caspase-3 activity (Figure 3E) and induced mitochondrial depolarization (JC-1 green monomers intensity increase, Figure 3F). Moreover, increased ratio of TUNEL-positive cell nuclei indicated apoptosis activation in miR-765-overexpresed ovarian cancer cells (Figure 3G). Thus miR-765 overexpression induced significant apoptosis activation in ovarian cancer cells. The non-sense miRNA control, lv-miRC, failed to induce significant apoptosis activation (Figure 3A–3G).

### miR-765 inhibition upregulates BRD4 and promotes ovarian cancer cell proliferation

**Figure 4:**
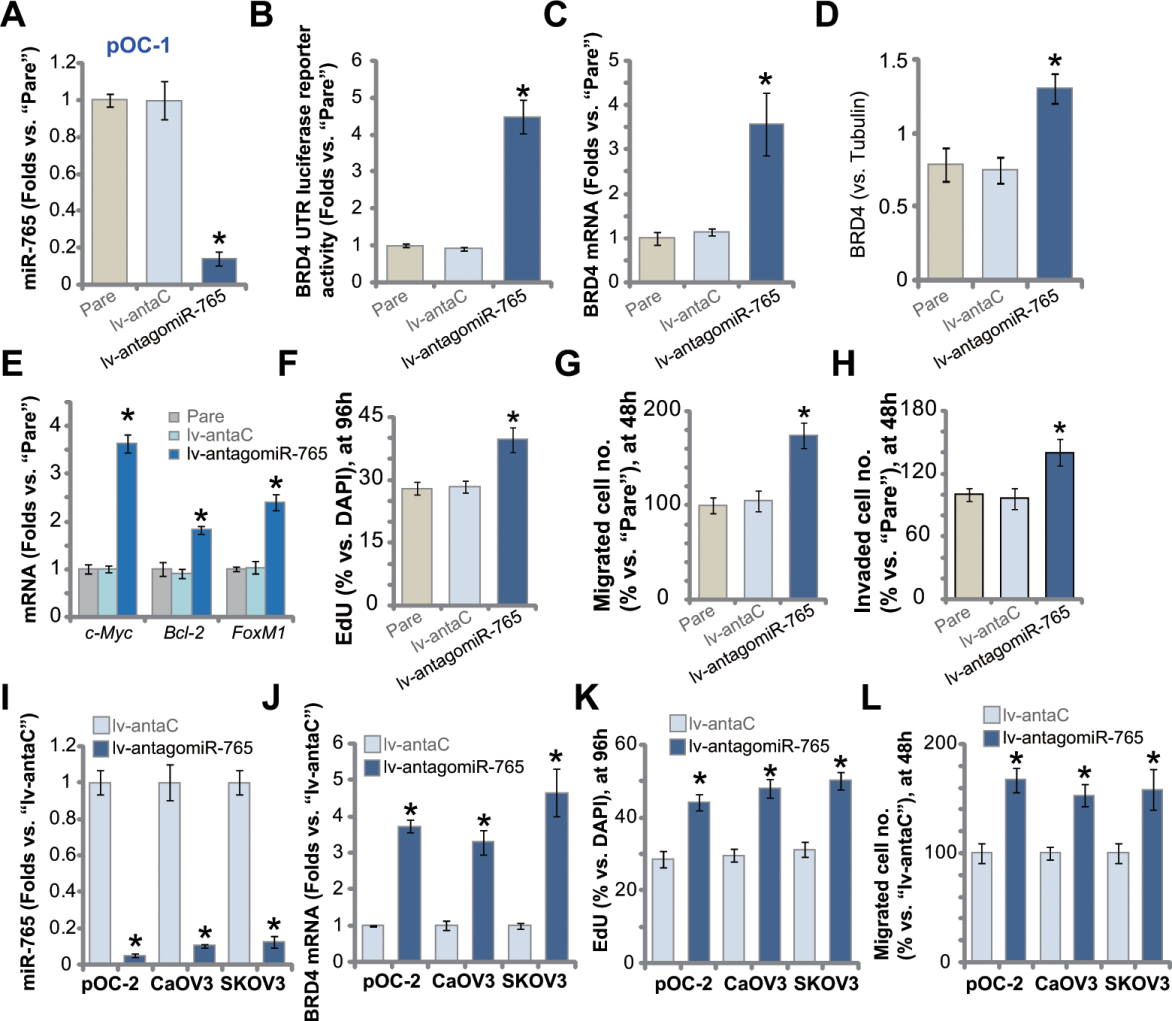
**miR-765 inhibition upregulates BRD4 and promotes ovarian cancer cell proliferation.** Primary ovarian cancer cells (pOC-1 and pOC-2) (**A**–**L**) or the established cell lines (CaOV3 and SKOV3) (**I**–**L**) were transduced with the lentiviral construct encoding the anti-sense of miR-765 precursor (lv-antagomiR-765) or control anti-sense sequence (lv-antaC), stable cells were established with selection by puromycin. Expression of miR-765 and listed genes was tested by qPCR and Western blotting assays (**A**, **C**–**E**, **I**, **J**), with results quantified; the relative BRD4 UTR luciferase reporter activity was tested as well (**B**). Cell proliferation (by counting EdU-positive nuclei ratio, **F**, **K**), cell migration (“Transwell” assays, **G**, **L**) and invasion (“Matrigel Transwell” assays, **H**) were tested, with results quantified. “Pare” stands for the parental control cells. For each assay, n=5 (five replicate well/dishes). Data were presented as mean ± standard deviation (SD). * *p* < 0.05 vs. “lv-antaC” cells. Experiments in this figure were repeated five times with similar results obtained.

We next studied whether miR-765 inhibition could exert opposite functions. A lentiviral construct encoding miR-765 anti-sense, lv-antagomiR-765, was established and transduced to pOC-1 primary cells. Stable cells were established with selection by puromycin-containing medium. qPCR assay results in Figure 4A demonstrated that miR-765 levels downregulated over 80% in lv-antagomiR-765-expresing pOC-1 cells. In contrast, the BRD4 3’-UTR luciferase reporter activity was significantly augmented (Figure 4B). Moreover, miR-765 inhibition by lv-antagomiR-765 induced upregulation of *BRD4* mRNA (Figure 4C) and protein (results were quantified in Figure 4D) in pOC-1 cells. BRD4-dependent genes, *c-Myc*, *Bcl-2* and *FoxM1*, were upregulated as well (Figure 4E).

Functional studies demonstrated that with BRD4 upregulation, lv-antagomiR-765-expressing pOC-1 cells showed enhanced cell proliferation (EdU-positive nuclei ratio increase, Figure 4F). In addition, lv-antagomiR-765 augmented pOC-1 cell migration (Figure 4G) and invasion (Figure 4H), tested by “Transwell” and “Matrigel Transwell” assays, respectively. In pOC-2 cells and established cell lines (SKOV3 and CaOV3), stable expression of lv-antagomiR-765 similarly resulted in robust miR-765 downregulation (Figure 4I). Conversely, *BRD4* mRNA levels were elevated (Figure 4J). Lv-antagomiR-765-induced miR-765 inhibition augmented proliferation (Figure 4K) and migration (Figure 4L) in the primary and established ovarian cancer cells. As expected, the lentiviral construct encoding control anti-sense sequence, lv-antaC, did not alter BRD4 expression and ovarian cancer cell functions (Figure 4A–4L). Collectively, miR-765 inhibition upregulated BRD4 and promoted ovarian cancer cell proliferation and migration.

### miR-765-induced anti-ovarian cancer cell activity is due to BRD4 silencing

**Figure 5:**
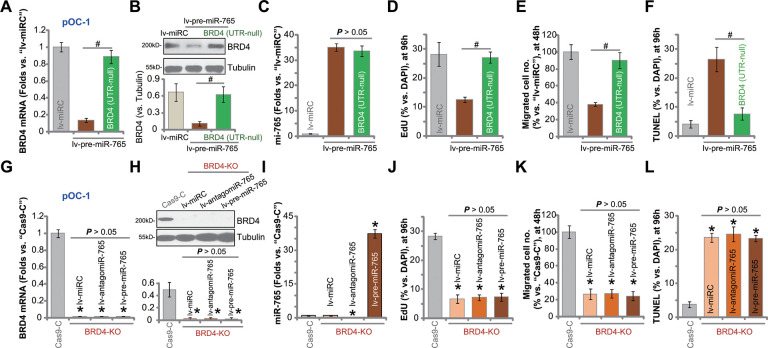
**miR-765-induced anti-ovarian cancer cell activity is due to BRD4 silencing.** The lv-pre-miR-765-expressing pOC-1 cells (Stb-L1) were further transduced with or without an UTR-null BRD4 construct [BRD4 (UTR-null)], stable cell were established; cells with the scramble non-sense miRNA (lv-miRC) were utilized as control cells. Expression of *BRD4* mRNA (**A**) and protein (**B**) as well as miR-765 (**C**) was shown; cells were further cultured for applied time periods, cell proliferation, migration and apoptosis were tested by EdU staining (**D**), “Transwell” (**E**) and TUNEL staining (**F**) assays, respectively, with results quantified (**D**–**F**). Stable pOC-1 cells expressing the lenti-CRISPR/Cas9-BRD4-KO construct, or the BRD4 KO cells, were further transduced with lv-pre-miR-765, miR-765 precursor anti-sense construct lentivirus (lv-antagomiR-765) or lv-miRC. Control cells were transfected with lenti-CRISPR/Cas9 control construct (“Cas9-C”), and stable cells established via selection by puromycin. Expression of *BRD4* mRNA (**G**) and protein (**H**) as well as miR-765 (**I**) was shown; cells were further cultured for applied time periods, cell proliferation (**J**), migration (**K**) and apoptosis (**L**) were tested similarly. For each assay, n=5 (five replicate well/dishes). Data were presented as mean ± standard deviation (SD). ^#^ *p* < 0.05 (**A**–**F**). * *p* < 0.05 vs. “Cas9-C” cells (**G**–**L**). Experiments in this figure were repeated five times with similar results obtained.

In order to confirm that BRD4 silencing caused miR-765-induced anti-ovarian cancer cell activity, an UTR-null BRD4 construct was transfected to lv-pre-miR-765-Stb-L1 pOC-1 cells (see Figures 1–3). As shown, after transfection of the construct [BRD4 (UTR-null)], *BRD4* mRNA (Figure 5A) and protein (Figure 5B) expression was restored in miR-765-overexpressed pOC-1 cells. However, miR-765 expression was unchanged by the UTR-null BRD4 construct (Figure 5C). Functional studies showed that lv-pre-miR-765-induced proliferation inhibition (EdU-positive nuclei ratio decrease, Figure 5D), migration suppression (Figure 5E), and cell apoptosis (TUNEL-positive nuclei ratio increase, Figure 5F) were largely attenuated byBRD4 (UTR-null) construct (Figure 5D–5F). Thus BRD4 silencing should be the primary reason of miR-765-induced anti-ovarian cancer cell activity.

To further support our hypothesis, a lenti-CRISPR/Cas9-BRD4-KO construct was transduced to pOC-1 cells. Stable cells with the construct, or BRD4-KO cells, were established. These cells had completely depleted *BRD4* mRNA (Figure 5G) and protein (Figure 5H). CRISPR/Cas9-induced BRD4 KO did not alter miR-765 expression (Figure 5I), but largely inhibited pOC-1 cell proliferation (Figure 5J) and migration (Figure 5K), while inducing apoptosis activation (Figure 5L). Thus, mimicking lv-pre-miR-765-induced actions, BRD4 KO exerted significant anti-ovarian cancer cell activity. In BRD4-KO cells, exogenously alteringmiR-765 expression, by infection withlv-pre-miR-765 or lv-antagomiR-765 (Figure 5I), did not change BRD4 expression (Figure 5G, 5H) or cellular functions including proliferation, migration and apoptosis (Figure 5J–5L). These results implied that miR-765 overexpression or inhibition was completely ineffective in BRD4 KO ovarian cancer cells, again confirming that BRD4 silencing caused miR-765-induced anti-ovarian cancer cell activity.

### miR-765 is downregulated in ovarian cancer tissues and cells

**Figure 6:**
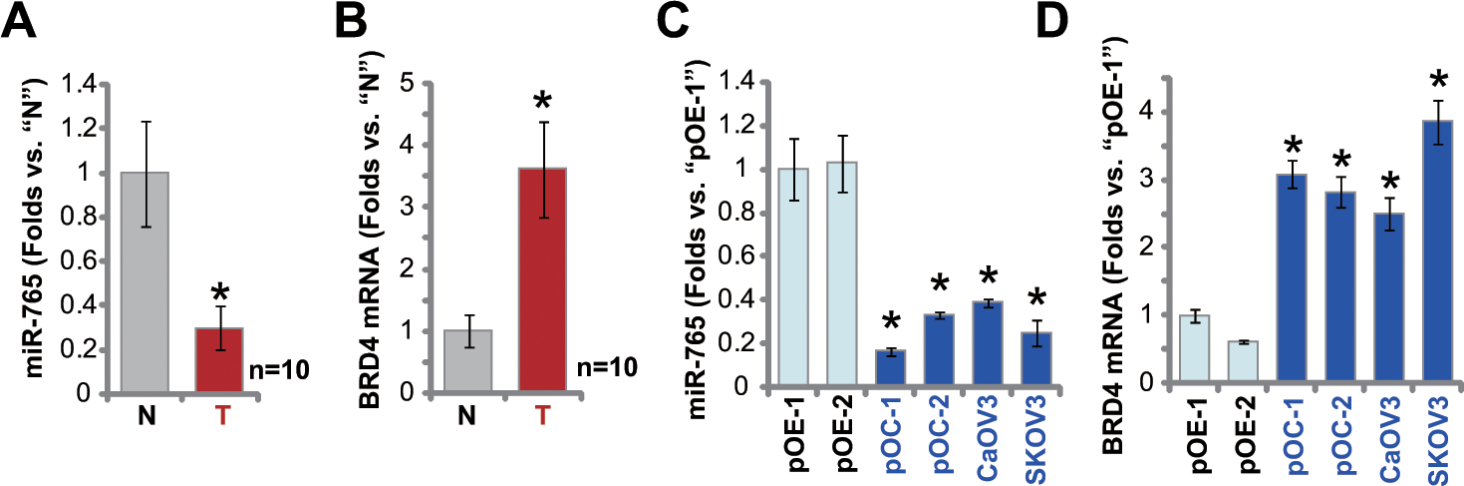
**miR-765 is downregulated in ovarian cancer tissues.** Expression of miR-765 and *BRD4* mRNA in ten (n=10) different ovarian cancer tissues (“T”) and surrounding paracancerous normal tissue (“N”) (**A**, **B**) as well as in primary ovarian cancer cells (pOC-1 and pOC-2), established ovarian cancer cell lines (CaOV3 and SKOV3), and human ovarian epithelial cells (pOE-1 and pOE-2) (**C**, **D**) was shown, with results quantified. Data were presented as mean ± standard deviation (SD). ^#^ *p* < 0.05 “N” tissues (**A**, **B**). * *p* < 0.05 vs. “pOE-1” cells (**C**, **D**). Experiments in this figure were repeated three times with similar results obtained.

At last, we tested expression of miR-765 in human ovarian cancer tissues. A set of 10 (n=10) different ovarian cancer tissues (“T”) and surrounding paracancerous normal tissues (“N”) were obtained and tested. As shown, miR-765 expression levels in the cancer tissues were significantly lower than those in the normal tissues (Figure 6A). In the contrast, *BRD4* mRNA expression was significantly elevated in cancer tissues (Figure 6B). When compared to miR-765-*BRD4* mRNA expression in primary human ovarian epithelial cells (pOE-1 and pOE-2), miR-765 downregulation (Figure 6C) and *BRD4* mRNA upregulation (Figure 6D) were observed in primary ovarian cancer cells (pOC-1 and pOC-2) and established cell lines (CaOV3 and SKOV3). These results showed that miR-765 is downregulated in ovarian cancer tissues and cells, and it is correlated with *BRD4* mRNA upregulation.

## DISCUSSION

Emerging studies have implied that miR-765 participated in cancer progression [23–27]. Yan et al., showed that ectopic overexpression of miR-765 inhibited osteosarcoma cell proliferation and migration [24]. Conversely, long noncoding RNA (LncRNA) Linc00511 had opposite effects by sponging miR-765 [24]. Ding et al., found that miR-765 exerted tumor-suppressive activity in tongue squamous cell carcinoma cells by targeting LAMC2 (laminin subunit gamma 2). Conversely, LINC00511 sponged miR-765 and abolished its activity [25]. Zheng et al., found that TNF-α increased miR-765 expression to inhibit oral squamous cancer cell migration [26]. By downregulating proteolipid protein 2 (PLP2), miR-765 eliminated lipids in clear cell renal cell carcinoma and acted as a tumor suppressor [23]. Xie et al., however, have proposed an oncogenic function of miR-765: It is upregulated inhuman hepatocellular carcinoma tissues and it promotes HCC cell proliferation by downregulating INPP4B (Inositol polyphosphate 4-phosphatase type II) [27].

We discovered that miR-765 could be a novel BRD4-targeting miRNA. In ovarian cancer cells, miR-765 mainly located at the cytosol fraction where it directly associated with *BRD4* mRNA. Ectopic overexpression of miR-765 by lv-pre-miR-765 robustly inhibited BRD4 3’-UTR luciferase reporter activity and significantly downregulated BRD4 expression in primary and established ovarian cancer cells. Conversely, lv-antagomiR-765-induced miR-765 silencing had opposite effects and it upregulated BRD4 expression in ovarian cancer cells. Interestingly, the two mutant miR-765 mimics with mutations at the binding sites to BRD4 3’-UTR failed to alter BRD4 expression in ovarian cancer cells. Importantly, in human ovarian cancer tissues, miR-765 was downregulated and negatively correlated with *BRD4* mRNA upregulation. Thus, miR-765 is a novel BRD4-targeting miRNA in ovarian cancer.

Ectopic expression of BRD4-targeting miRNA has proven to be a good strategy to inhibit BRD4-driven cancer progression. Zheng et al., reported that expression of miR-4651 silenced BRD4 to inhibit non-small cell lung cancer (NSCLC) cell growth and proliferation [28]. Li et al., found that miR-608 induced pancreatic ductal adenocarcinoma cell apoptosis by silencing BRD4 [29]. Similarly, BRD4-targeting miR-608 inhibited HCC cell proliferation [30]. Kang and colleagues found that targeting BRD4 by miR-612 inhibited malignant development of NSCLC cells [31]. In cutaneous T-cell lymphoma, miR-29b downregulation is associated with BRD4-mediated activation of several oncogenes [32].

Here in primary ovarian cancer cells and established cell lines (SKOV3 and CaOV3), ectopic overexpression of miR-765 by lv-pre-miR-765 inhibited cell proliferation, migration and invasion, and simultaneously induced cell apoptosis activation. In contrast, lv-antagomiR-765-induced miR-765 inhibition augmented ovarian cancer cell proliferation, migration and invasion. Significantly, miR-765 overexpression-induced anti-ovarian cancer cell activity was largely inhibited by restoring BRD4 expression using the UTR-null BRD4. In addition, mimicking lv-pre-miR-765’s actions, CRISPR/Cas9-induced BRD4 KO induced significant proliferation inhibition and apoptosis in ovarian cancer cells. Importantly, BRD4 KO in ovarian cancer cells abolished the functional impact of miR-765. Therefore, miR-765 targeted and silenced BRD4 to potently inhibit ovarian cancer cell progression.

## MATERIALS AND METHODS

### Chemicals and reagents

Puromycin, polybrene, the anti-BRDT antibody, fetal bovine serum (FBS) and other cell culture reagents were provided by Sigma-Aldrich Chemicals (St Louis, MO, USA). Other antibodies utilized in this study were purchased from Cell Signaling Tech China (Shanghai, China). From Genechem Co (Shanghai, China), primers, nucleotide sequences, constructs and viruses were obtained. RNA assay reagents were provided by Thermo-Fisher Invitrogen (Beijing, China).

### Human tissues

From ten (10) primary ovarian cancer patients (all with written-informed consent, Stage II-III), ovarian cancer tissues (“T”) and para-cancer normal ovarian epithelial tissues (“N”) were obtained. Fresh human tissue specimens were washed, minced, homogenized in tissue lysis buffer (Beyotime Biotechnology, Wuxi, China). Experiments using human tissues and cells were approved by the Ethics Board of Soochow University, according to the Declaration of Helsinki.

### Cells

Primary human ovarian epithelial cells, pOE-1 and pOE-2 were provided by Dr. Bi [33]. Cells were cultured in MCDB109/M199 medium plus 15% FBS. To obtain primary human ovarian cancer cells, fresh ovarian cancer tissues were washed, minced into small pieces, and digested with Collagenase I (Sigma-Aldrich) and DNase (Sigma-Aldrich). The resulting single-cell suspensions were pelleted and washed. Fibroblasts, blood vessel cells, and immune cells were abandoned. Purified cancer cells were cultured in the described medium [34]. Two different primary ovarian cancer cells, pOC-1 and pOC-2 were established. Established ovarian cancer cell lines, SKOV3 and CaOV3, were purchased from Cell Bank of Chinese Academy of Sciences (Shanghai, China). Cells were cultured in RPMI-1640 medium supplemented with 10% FBS.

### Quantitative reverse transcription–polymerase chain reaction (qPCR)

Total cellular RNA was isolated from human tissues and cells using a RNA Kit (TIANGEN, Beijing, China). For RNA quantification, a NanoDrop™ 2000 Spectrophotometer (Invitrogen Thermo Fisher Scientific, Shanghai, China) was utilized. The PrimeScript RT Master Mix (Perfect Real Time; Takara Bio, Tokyo, Japan) was utilized for reverse transcription to generate complementary DNA (cDNA). TB Green Premix Ex Taq™ II (Takara Bio, Inc, Beijing, China) was utilized to test expression of targeted genes under the ABI 7900 system (Applied Biosystems, Foster City, CA). *GAPDH* was tested as the internal reference gene. A miRcute Plus miRNA qPCR Kit (SYBR Green; TIANGEN) was used for testing miR-765 expression, the result was normalized to U6 small nuclear RNA. All data were quantified through the 2^−ΔΔCt^ method. mRNA primers for *BRD4*, *U6*, *GAPDH*, *c-Myc*, *Bcl-2* and *FoxM1* were provided by Dr. Zheng [28]. MiR-765 primers, F: TGGAGGAGAAGGAAGGTG and R: GAACATGTCTGCGTATCTC, were provided by Genechem (Shanghai, China).

### Subcellular fractionation location

A Cytoplasmic and Nuclear RNA Purification Kit (Norgen, Belmont, CA, USA) was utilized for the isolation of RNA from the cytoplasmic and nuclear fractions based on the attached protocols.

### miR-765 overexpression or inhibition

The nucleotide sequences encoding the miR-765 precursor (pre-miR-765, Using the primers F: TGGAGGAGAAGGAAGGTG and R: GAACATGTCTGCGTATCTC) and miR-765 anti-sense were designed and synthesized by Genechem. Thereafter, the two were individually sub-cloned into GV369 constructs (Genechem). Each construct, along with the lentivirus-packing helper plasmids (Genechem), were co-transfected to HEK-293T cells. Afterwards, pre-miR-765-expressing lentivirus (“lv-pre-miR-765”) and pre-miR-765 anti-sense lentivirus (“lv-antagomiR-765”) were generated. Viruses were added to cultured OS cells (maintained in polybrene-containing complete medium). To select stable cells puromycin (3.0 μg/mL) was added in the medium (for five passages). Mature miR-765 expression in stable OS cells was tested by qPCR. For transfection of miRNA mimics, cells were initially seeded into six-well plates and transfected with the applied miR mimic (500 nM, 36h) using Lipofectamine 3000.

### “Transwell” migration and invasion assays

For migration assays, OS cells with the applied genetic modifications were trypsinized, rinsed, centrifuged, and resuspended in serum-free medium. For each well, 200 μL cell suspension containing 3 × 10^4^ OS cells were added to the upper chamber of the “Transwell” inserts (BD Biosciences, Shanghai, China). The complete medium with 10% FBS was added to the lower chamber. After incubation, the non-migrated cells were removed. The migrated cells were fixed, stained and counted. Five visual fields of each condition were randomly selected and the average number of migrated cells was calculated. Invasion assays were conducted using the same protocol, only the “Transwell” inserts were pre-coated with Matrigel (BD Biosciences).

### Western blotting

OS cells were incubated with RIPA protein lysis buffer (TIANGEN) to isolate total cellular protein. A BCA Protein Assay Kit (TIANGEN) was utilized to quantify protein concentration. Equal amounts of protein extracts (40 μg proteins per lane) were separated by 10-12% sodium dodecyl sulfate-polyacrylamide (SDS-PAGE) gels and were transferred to PVDF blots (Millipore, Billerica, MA, USA). After blocking, blots were incubated with the applied primary antibodies overnight at 4° C and subsequently incubated with horseradish peroxidase (HRP)-conjugated secondary antibodies at room temperature for 1h. An Immobilon ECL Ultra Western HRP Substrate (Millipore) kit was utilized to detect the blotting signals.

### Transfection of miRNA mimics

OS cells were seeded into six-well plates at approximately 60% confluence. Lipofectamine 3000 protocol was utilized for the transfection of 500 nM of the wild-type (“WT”) or the mutant (“Mut”) miR-765 mimics (for 48h). Expression of miR-765 was determined by qPCR after transfection.

### BRD4 UTR luciferase reporter assay

The pMIR-REPORT Reporter vector (Ambion; Thermo Fisher Scientific, Shanghai, China) containing BRD4’s 3’-UTR sequence with the predicted miR-765 binding sites, or pMIR-BRD4-3ʹ-UTR, was provided by Dr. Zhao [28]. The construct was transfected to OS cells by Lipofectamine 3000. Cells were then infected with lv-pre-miR-765 or lv-antagomiR-765 for 48h. Thereafter, cells were harvested and assayed for the measurement of luciferase activity using a Dual-Luciferase Reporter Assay System (Promega, Shanghai, China).

### BRD4 knockout (KO)

A lenti-CRISPR/Cas9-BRD4-KO-GFP construct was provided by Dr. Zhao [35]. OS cells were initially seeded into six-well plates and transfected with the construct by Lipofectamine 3000. GFP-positive cells were sorted by FACS. Cells were then cultured in 96-well plates to form monoclonal cells. BRD4 knockout (KO) was screened by Western blotting and qPCR assays, and single stable cells were established.

### UTR-null BRD4

The lentiviral construct containing3’-UTR-null BRD4 was provided by Dr. Zheng [28]. The construct was transfected to cultured OS cells by Lipofectamine 3000. Expression of UTR-null BRD4 was verified by Western blotting and qPCR assays.

### RNA pull-down

A Pierce Magnetic RNA pull-down Kit was utilized for RNA pull-down assay through the described protocols [36, 37]. Briefly, OS cells were transfected with biotinylated miR-765 mimic or control mimic (Genechem, each at 300 nM) for 24h, and cells were harvested [37]. Cell lysates were incubated with streptavidin-coated magnetic beads to pull-down biotin-captured RNA complex [36]. The miR-765-bound *BRD4* mRNA was tested by qPCR, and its level was normalized to “Input” controls.

### Cell counting kit-8 (CCK-8)

A cell suspension (100 μL) containing 3 × 10^3^ viable OS cells with applied genetic modifications were seeded into each well of 96-well plates. Each group contained five replicates. Following incubation for 96h, 10 μL of CCK-8 solution (Dojindo Laboratories, Rockville, MD, USA) was added into each well for 2h. CCK-8 absorbance was measured at 450 nm.

### Colony formation

Ovarian cancer cells were seeded into 10-cm dishes (at 1×10^4^ cells per dish). Complete medium was renewed every two days. After 10 days, the number of large colonies was counted.

### EdU incorporation

Cells were seeded into 96-well plates at 3, 000 cells per well and cultured for applied time periods. Afterwards, cells were cultured for 2h with EdU medium diluents. Cells were then incubated with DAPI solution for15 min, followed by observation under the fluorescence microscope (Olympus, Tokyo, Japan).

### TUNEL assay

OS cells with applied genetic treatments were treated with 2% formaldehyde and 0.1% Triton X-100 for 5 min. Cells were then incubated with TUNEL reaction buffer for 1h and co-stained with DAPI. Apoptotic cells were visualized under Olympus microscope.

### Caspase-3 activity

As described before [38], total cellular lysates (20μg of each treatment) were incubated with 7-amino-4-trifluoromethylcoumarin (AFC)-conjugated caspase-3 substrate. After 3h incubation, the AFC activity was tested through the Infinite 200 PRO reader at 400 nm excitation and 505 nm emission.

### Histone DNA ELISA assay

A cell suspension (200 μL) containing 3 × 10^3^ viable OS cells with applied genetic modifications was seeded into each well of 96-well plates. Cells were cultured for 72h and a Histone DNA ELISA kit was utilized to test Histone-bound DNA contents [39].

### Mitochondrial depolarization

Ovarian cancer cells with the indicate genetic treatments were seeded into six-well plates (1 ×10^5^ cells per well). JC-1 staining of mitochondrial membrane potential was described previously [40, 41]. JC-1 green monomer intensity (at 488 nm) was measured.

### Statistical analysis

Data were presented as the mean ± standard deviation (SD). The Student’s *t*-test (Excel 2007) was utilized for the comparison between two specific treatment groups. Alternatively, one-way analysis of variance (ANOVA) with Tukey’s test was utilized to test difference between multiple groups (SPSS23.0, SPSS Co. Chicago, CA). *p* values <0.05 were considered statistically significant.
